# Methods for 20S Immunoproteasome and 20S Constitutive Proteasome Determination Based on SPRI Biosensors

**DOI:** 10.1007/s12195-017-0478-7

**Published:** 2017-01-24

**Authors:** Sankiewicz Anna, Markowska Agnieszka, Lukaszewski Zenon, Puzan Beata, Gorodkiewicz Ewa

**Affiliations:** 1 Department of Electrochemistry, Institute of Chemistry, University of Bialystok, Ciolkowskiego 1K, 15-245 Bialystok, Poland; 2 Department of Organic Chemistry, Medical University of Bialystok, Kilinskiego 1, 15-089 Bialystok, Poland; 3 Faculty of Chemical Technology, Poznan University of Technology, pl. Sklodowskiej-Curie 5, 60-965 Poznan, Poland

**Keywords:** 20S proteasome, Proteasome inhibitors, ONX 0914, PSI, Sensor, Surface plasmone resonance imaging

## Abstract

The 20S proteasome, released into the circulation, is a novel cancer biomarker. It exists in two forms: the constitutive proteasome (20Sc) and the immunoproteasome (20Si), which both have separate diagnostic significance. The aim of this work was to develop new methods for 20Si and 20Sc determination. Five alternative specific biosensors usable with the surface plasmon resonance imaging (SPRI) technique for 20Si determination have been developed. Specific 20Si entrapment on the biosensor surface from an analyzed solution was achieved by means of an immobilized specific 20Si receptor. Four of the biosensors contain newly synthesized specific 20Si receptors, while the fifth contains the inhibitor ONX 0914. A method for 20Sc determination using an SPRI biosensor containing PSI inhibitor has been developed. By the introduction of an inhibitor blocking 20Si, 20Sc is selectively determined. All of the methods developed for 20Si and 20Sc determination exhibit good selectivity and satisfactory precision, recoveries and dynamic response ranges. 20Si and 20Sc were determined in blood plasma samples from healthy donors and patients with acute leukemia. In the case of these patients 20Si was the major component, and its level was more than one order of magnitude higher than in the healthy donors.

## Introduction

The 20S proteasome (20S), released into the circulation, is a novel biomarker for the prognosis and monitoring of patients suffering from various types of cancers, as well as other pathologies.^27^ Patients with acute lymphoblastic (ALL) and myeloblastic leukemias (AML) exhibit 20S levels in blood serum elevated by 1.5 orders of magnitude in comparison with the control.^10^ However, 20S occurs in two forms, the constitutive proteasome (20Sc) and the immunoproteasome (20Si), and the determination of both forms is desirable. In the cases of ALL and AML, 20Si is the dominant sub-form of 20S.^18^ Apart from acute leukemia, 20Si plays a role in the pathogenesis of autoimmune diseases and neuropathologies.^1^,^17^,^18^ The upregulation of immunoproteasome is a response to challenges that induce stress and injury.^6^ In particular, the immunoproteasome plays a role in the adaptive immune response and is more efficient at eliciting responses than the constitutive proteasome.^4^

Inhibition of 20Sc and 20Si is an efficient therapy in the treatment of hematological malignancies.^20^ Selective inhibitors of 20Si or 20Sc, as well as inhibitors of both forms, are used.

Both 20Sc and 20Si are barrel-shaped (MW 700 kDa) and composed of four rings. Each of the rings contains seven distinct subunits. There are two identical outer α rings and two inner β rings. The α-rings control the entry of the substrate proteins into the central catalytic chamber and bind the regulators. The β rings contain three different catalytic sites. In the case of 20Sc, the β1, β2, and β5 subunits are responsible for caspase-like, trypsin-like and chymotrypsin-like activities, for cleavage of proteins after acidic, basic, and hydrophobic amino acids, respectively.^7^ In the case of 20Si, the β2i and β5i subunits act similarly to the β2 and β5 subunits in 20Sc. However, in the case of the β1i subunit (also known as LMP2—low molecular weight protein 2) the ability to cleave a protein after an acidic residue almost vanishes, and instead there appears an ability to act as a chymotrypsin-like subunit.^5^

Two methods have been used for 20Si determination: the enzyme-linked immunoabsorbent (ELISA) test^18^,^23^,^24^ and semiquantitative western blotting.^3^,^18^,^21^,^29^ These methods are broadly used in the determination of biologically active species. However, both methods are indirect “labeled methods”. The use of labels may be the reason for losing of the protein functional properties.

An alternative method for immunoproteasome detection may be a surface plasmon resonance imaging (SPRI) biosensor. This is a label-free, surface-sensitive spectroscopic technique used to examine the interaction between biomolecules.^13^,^28^ SPRI detects changes in the refractive index within a short distance from the surface of a thin metal film caused by molecules bound to the metal surface. The sensing surface usually consist of glass coated with a thin metal layer (e.g. gold) and a layer of active biomolecules as a receptor. The antibody or inhibitor can be used for capturing of an analyte from the solution.

SPRI biosensors have been successfully used for the determination of biologically active substances such as lysosomal proteases,^8^,^9^,^11^ 20S proteasome,^10^ ubiquitin carboxyl-terminal hydrolase L1 (UCHL1),^18^ aromatase,^1^ angiopoietin-2^26^ and transgelin-2 (TAGLN2).^15^ The determination of total 20S proteasome concentration, i.e. without selective determination of sub-types of 20S, has been reported.^10^ Several SPRI biosensor applications for clinical research have been reported.^9^,^12^,^16^,^25^

The aim of this work was to develop methods for the determination both 20Si and 20Sc i.e. both sub-forms of 20S proteasome. This is entirely new approach. The simplest method for 20Si determination was based on a specific biosensor, containing 20Si inhibitor as a receptor, with detection by the surface plasmon resonance imaging (SPRI) technique. In this work, the commercial inhibitor ONX 0914 was used for the construction of the biosensor. Additionally, new 20Si receptors were synthesized in order to obtain a substance of simple chemical structure suitable only for analytical purposes. A carboxyl group was introduced into the compound structure for immobilization of the receptor to a cysteamine linker. Inhibiting effect of synthesized receptors on the amidolytic activity of chymotrypsin and trypsin has been determined. These enzymes are active parts of 20Si and 20Sc. The analytical parameters of several optional SPRI biosensors were optimized and the biosensor’s ability to determine 20Si in biological samples was tested. Apart from direct 20 Si determination, the newly synthesized receptors were used for indirect determination of 20Sc i.e. the other form of 20S proteasome.

## Experimental

### Reagents

Fmoc-Phe-OH (Fmoc = 9-fluorenylmethyloxycarbonyl), chloranil, acetaldehyde, HOBt (1-hydroxybenzotriazole), Fmoc-nLeu-OH (Fmoc-2-APA-OH, APA 2 aminopentanoic acid), Fmoc-Ala-OH, Fmoc-Leu-OH, Fmoc-hPhe-OH (hPhe homoPhe, 4-phenylbutyric acid) HPLC solvent acetonitrile were purchased from Merck (Novabiochem, Darmstadt, Germany, www.merckmillipore.com/). TFA (trifluoroacetic acid), DIPEA (diisopropylethylamine), DIC (diisopropylcarbodiimide), piperidine, TBTU (O-(benzotriazol-1-yl)-N,N,N′,N′-tetramethyluronium tetrafluoroborate, NMP = 1-methyl-2-pyrrolidone and were obtained from Iris Biotech GmbH (all Marktrewitz, Germany, www.iris-biotech.de/). DCM (dichloromethane), DMF (dimethylformamide) and methanol were the products of Chempur (Piekary Slaskie, Poland, www.chempur.pl/). DCM was used without further purification. DMF was distillated over ninhydrin and stored under molecular sieves 4A. Trypsin, chymotrypsin, Suc-Phe-pNA and Bzl-l-Arg-pNA.HCl (Suc [succinyl], pNA [4-nitroanilide], Bzl [benzyl]) were purchased from Sigma (Schnelldorf, Germany, www.sigmaaldrich.com/). Phosphate buffered saline (PBS) were purchased from Lublin Label Vaccines (Lublin, Poland, www.biomed.lublin.pl/).

20S proteasome (mammalian) (AFFINITI Research Products Ltd, Mamhead, UK), commercial inhibitor for immunoproteasome (ONX 0914) (Houston, USA, www.selleckchem.com/), protein immunoproteasome (Basepoint Business Centre, United Kingdom, www.vivabioscience.com/), human albumin, cysteamine hydrochloride, N-ethyl-N′-(3-dimethylaminopropyl) carbodiimide (EDC) were purchased from Sigma (all Steinheim, Germany, www.sigmaaldrich.com). 1-octadecanothiol (ODM) and N-Hydroxysuccinimide (NHS) was obtained from Aldrich (Munich, Germany, www.sigmaaldrich.com). HBS-ES solution pH 7.4 (0.01 M HEPES, 0.15 M sodium chloride, 0.005% Tween 20, 3 mM EDTA), acetic buffer pH 3.79–5.57, phosphate buffer pH 7.17–8.04, carbonate buffer pH 8.50–9.86 (all Biomed, Lublin, Poland, www.biomed.pl/), photopolimer ELPEMER SD 2054, hydrophobic protective paint SD 2368 UV SG-DG (Peters, Kempen, Germany, www.peters.de/) were used as received. Aqueous solutions were prepared with MilliQ water (Simplicity^®^MILLIPORE).

### Biological Samples

Plasma samples from healthy adult donors (n = 4) and patients with acute lymphoblastic leukemia (n = 5) were supplied by the Department of Hematology, Medical University of Bialystok, Poland. Plasma samples from patients with acute lymphoblastic leukemia were diluted 10 times with PBS.

All of the samples were provided after obtaining the consent of the Local Bioethical Commission for the study of the biological material collected.

## Procedures

### Peptide Synthesis

The peptides shown in Table 1 were synthesized manually using the standard Fmoc-based strategy.^2^ The first Fmoc amino acid was loaded onto the 2-chlorotrityl chloride resin with a twofold molar excess of DIPEA in DCM. Fmoc deprotection steps were carried out with 20% (v/v) piperidine in DMF/NMP (1:1, v/v) for 15 min. The coupling reactions of Fmoc amino acids were performed in DMF/NMP/DCM (1:1:1) using a molar ratio of amino acid/DIC/HOBt/resin of 3:3:3:1. The reactions were monitored with the Steward chloranil test.^30^ Cleavage from the resin was carried out with TFA/water (95/5). After 2.5 h stirring, the resin was filtered and washed with TFA. The combined filtrates were concentrated under reduced pressure. The crude peptide was precipitated and washed with cold diethyl ether, filtered, dissolved in water and lyophilized.

**Table 1:** Synthesized compounds and their basic characteristics.

No.	Peptide	Yield (%)	Retention time (min)	MW	[M+H]^+^
1	H-Phe-Leu-Phe-OH	52	12.80	425.5	426.4
2	H-Phe-Phe–Phe-OH	61	13.03	459.5	460.2
3	H-Phe-Ala-Phe-OH	68	11.22	383.4	384.1
4	H-Leu-Phe–Phe-OH	62	10.81	425.5	426.5
5	H-Phe-Leu–Leu-OH	58	10.57	391.5	392.5
6	H-Phe-Phe-Leu-OH	59	10.61	425.5	426.4
7	H-Phe-hPhe-Phe-OH	42	11.81	473.6	474.5
8	H-Leu-Leu-Phe-OH	53	10.67	391.5	392.7
9	H-Phe-Phe-OH	69	9.89	312.4	313.5
10	H-Leu-Phe-Leu-OH	59	10.34	391.5	392.2
11	H-Phe-Leu-nLeu-OH	52	9.98	391.5	392.9

The Waters system (Waters S.A.S., BP 608, 78056 Saint-Quentin, En Yvelines Cedex, France) was used for analytical and semipreparatory HPLC (Phenomenex C18, Jupiter 90A, 4 micron, 250 × 10 mm; Phenomenex C18, Jupiter 300A, 5 micron, 250 × 4 mm; solvents: A, 0.1% aqueous TFA; B, 0.1% TFA in acetonitrile, gradient 0–100% B in A in 30 min, analytical flow rate 1 mL min^−1^, preparatory flow rate 1.5 mL min^−1^ monitored at 220 nm). In the semipreparatory HPLC the major peak fraction was pooled and lyophilized. Molecular weight determination was performed by mass spectrometry using a Bruker Daltonics Esquire 6000 (Bruker Daltonik GmbH, Leipzig, Germany) with electrospray ionization (ESI).

### Enzymatic Investigations

Determination of amidolytic activity was performed as described by Okada.^22^ The synthetic amidolytic (chromogenic) substrates (Bzl-l-Arg-pNA.HCl for trypsin and Suc-Phe-pNA for chymotrypsin) are selected to give appropriate specificity for inhibition of the serine proteases. A chromophore (para-nitroaniline = pNA, yellow color) is cleaved from the C-terminus amide bond by the protease, and the absorbance can be detected.

A detailed description of the method is given below. The buffer and the enzyme solution contained:

- borane buffer—0.5 mL (pH 7.5), enzyme: trypsin (0.4 units mL^−1^), synthetic substrate: Bzl-l-Arg-pNA.HCl (0.2 mL, 8 mM);
- tris buffer—0.6 mL (pH 9.0), enzyme: chymotrypsin (0.4 units mL^−1^), synthetic substrate: Suc-Phe-pNA (0.2 mL, 8 mM).

The buffer and 0.1 mL of enzyme solution were added to 0.2 mL of the examined compound dissolved in 0.15 M NaCl (1–11) (as control 0.15 M NaCl). The mixture was incubated for 3 min at 37 °C, then the synthetic substrate was added. After 20 min of incubation, the reaction was stopped by adding 0.1 mL of 50% acetic acid, and the absorbance of the released p-nitroaniline was measured at 405 nm (Spekol 1300, Analytic Jena). Every value represents the average of triplicate determination. The IC_50_ value was taken as the concentration of the inhibitor which decreased the absorbance at 405 nm by 50% compared with the absorbance measured under the same conditions without the inhibitor. The IC_50_ values obtained for the inhibitors are given in Table 2.

**Table 2:** Inhibiting effect of synthesized peptides on the amidolytic activity of enzymes.

No	Peptide	IC_50_ mM	
		Trypsin	Chymotrypsin
1	PheLeuPhe	–	0.178 ± 0.014
2	PhePhePhe	–	0.185 ± 0.014
3	PheAlaPhe	–	0.187 ± 0.014
4	LeuPhePhe	–	0.184 ± 0.014
5	PheLeuLeu	–	0.184 ± 0.014
6	PhePheLeu	–	0.174 ± 0.013
7	PhehomoPhePhe	0.044 ± 0.003	0.185 ± 0.014
8	LeuLeuPhe	0.079 ± 0.006	0.152 ± 0.012
9	PhePhe	–	0.141 ± 0.011
10	LeuPheLeu	12 ± 0.96	1.83 ± 0.15
11	PheLeu-norLeu	18 ± 1.44	1.92 ± 0.15

All of the tripeptides obtained exhibit chymotrypsin inhibition.

### Chip Preparation

Gold chip were manufactured as described in a previous paper.^11^ The gold surface of the chip was covered with photopolymer and hydrophobic paint.^8^ The chip has nine places each with 12 free gold surfaces. Using this chip, nine different examined solutions can be simultaneously measured without mixing the tested solutions. Twelve single SPRI measurements can be performed from one solution.

### AFM Measurements

Atomic Force Microscopy (AFM) enables observation of the surface of the biosensor during its preparation. AFM measurements were performed to confirm the creation of successive layers. All measurements were performed using a commercial Ntegra Prima scanning probe microscope (NT-MDT, Russia) in tapping mode in ambient conditions. Ethalon probes (NT-MDT, Russia), with resonance frequency around 140 kHz and curvature radius less than 10 nm, were used.

### ONX 0914 Immobilization

**Figure 1:**
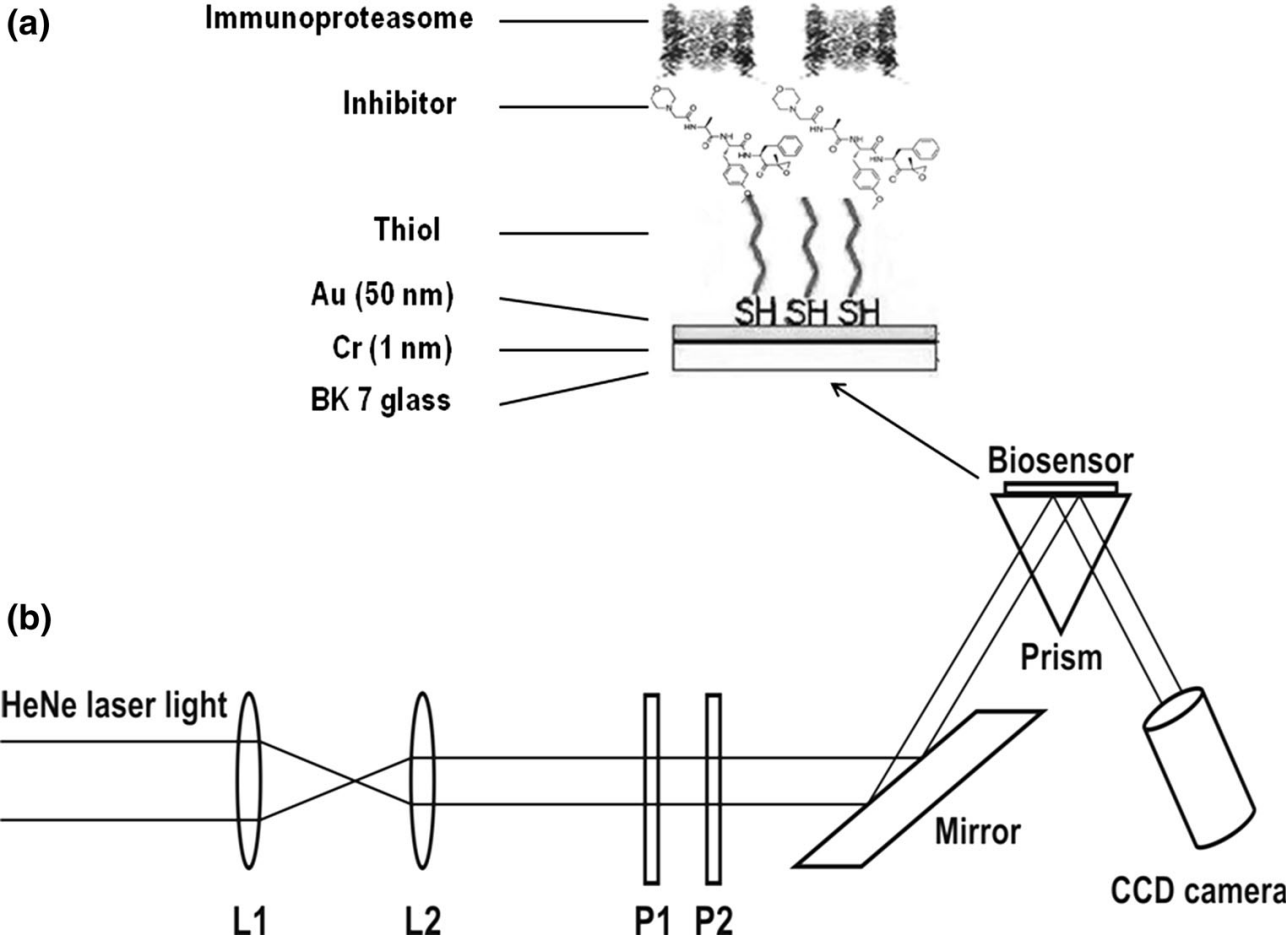
Schematic illustration of the sensor’s active part (a) and the apparatus (b).

Chips were immersed in an ethanolic solution 1-octadecanothiol (ODM) (20 nM) for 24 h at room temperature. Then the biosensors have been repeatedly washed in ethanol and water, and dried under a stream of argon. Chips prepared in this way can be stored and used for subsequent measurements. The commercial inhibitor ONX 0914 was then applied to the active sites of the biosensor and incubated for 24 h at room temperature. After that time, the process of immobilization of the inhibitor by hydrophobic interaction was completed. A biosensor prepared in this way should be used directly for analysis. A diagram of the chip appears in Fig. 1a.

### Newly Synthesized Receptors Immobilization

The chip was immersed in a vessel with an ethanolic solution of cysteamine for 2 h at room temperature. After 2 h, the chip was rinsed with ethanol and water and dried under a stream of argon. A chip prepared in this manner can be stored and used for subsequent measurements. Each of the synthesised inhibitor solutions was activated with NHS (250 mM) and EDC (250 mM) in the ratio 1: 1 and carbonate buffer (pH 8.5) as a suitable reaction medium. Next, 3 *μ*L of this solution was placed on each of nine active sites on the amine-modified surface. The closed vessel with the biosensor and under appropriate conditions of humidity was incubated at 37 °C for 1 h. Next, the biosensor was rinsed with water and dried under a stream of argon. A biosensor prepared in this way should be used directly for testing and analysis.

AFM measurements were performed to confirm the creation of subsequent layers: gold, thiol (cysteamine or ODM), inhibitor and immobilized immunoproteasome 20S. All measurements were done in ambient conditions. The AFM pictures confirm that the described stages of the creation of each layer on the biosensor surface has really take place. This may be concluded on the basis of the creation of different structures after each stage.

### SPRI Measurements

SPRI measurements were performed on a home-made apparatus which has been successfully used and described previously.^8^^–^ 11 The apparatus (Fig. 1b) consists of a HeNe laser (JDS Uniphase, Edmund Industrial Optics, USA), two glass lenses L1 (f = 3 mm) and L2 (f = 300 mm), two polarizers (P1 and P2), a mirror, a prism and a CCD camera (QICam, QImaging, Canada). NIH Image J version 1.42 software was used for evaluation of the SPRI images in 2D form.

SPRI measurements for the protein biosensor array were performed as described elsewhere.^8^,^11^ Briefly, the measurements were performed at a fixed angle of incident light and the reflectivity was simultaneously measured cross an entire chip surface. The contrast values obtained for all pixels across a particular sample single spot were integrated. The signal was measured twice on the basis of registered images, after immobilization of the inhibitor and after immunoproteasome interaction. The SPRI signal, which is proportional to the coupled protein, was obtained by subtraction between the signals before and after interaction with protein for each spot separately.

A background correction was applied i.e. some of the sites on the biosensor covered with PBS buffer were used as a control. Non-specific binding was monitored by measuring the SPRI signal at a site on the chip without the receptor (ligand). Minimization of non-specific binding was achieved by preparing samples in PBS buffer (NaCl an KCl concentration ~200 mM) at pH 7.4 near the isoelectric point of the protein.

In the case of indirect 20Sc determination, the inhibitor (ONX 0914 or tripeptide) solution was introduced into the analyzed solution (blood plasma or blood plasma diluted with PBS buffer) in the volume necessary to achieve a concentration of 15 *μ*g mL^−1^.

## Results and Discussion

### Biosensor Based on ONX 0914 Inhibitor

The biosensor was made by immobilization of the inhibitor via ODM linker. The ODM was anchored onto the gold surface by a linker thiol group. ONX 0914 was immobilized due to hydrophobic interaction between the octadecyl group of ODM and the inhibitor, as is described above (*ONX 0914 immobilization*).

### Optimization of ONX 0914 Concentration

**Figure 2:**
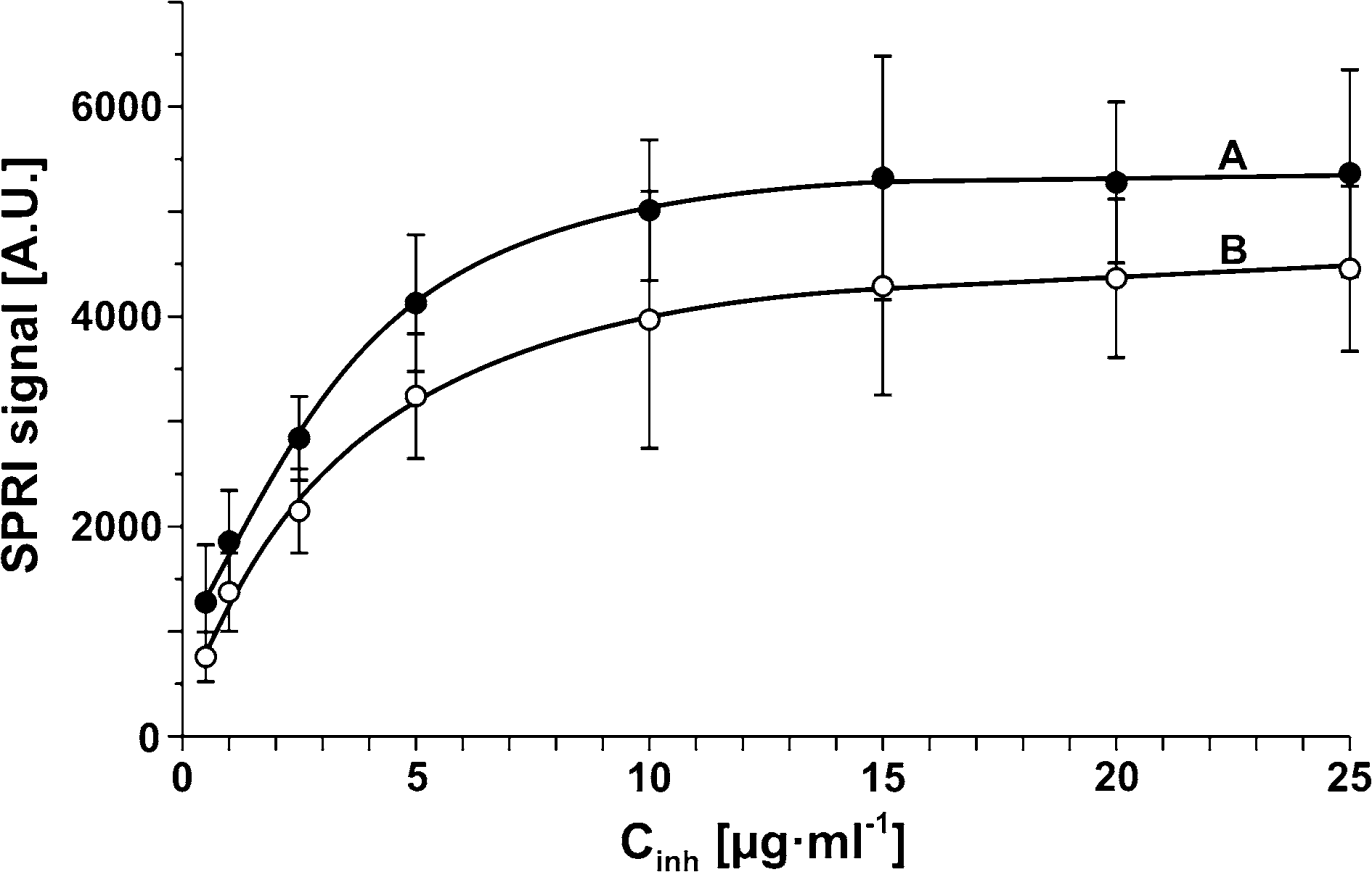
Dependence of SPRI signal on receptor concentration: (a) ONX 0914 inhibitor, (b) inhibitor 5. 20Si concentration: 2.8 *μ*g mL^−1^, pH 7.4.

Concentration of receptor is a significant parameter at SPRI measurement, and ought therefore to be optimized. At constant concentration of the analyte to-be-determined, the dependence of the analytical signal as a function of receptor concentration exhibits a Langmuirian shape with formation of a plateau. Correct SPRI measurement should be performed with the receptor concentration corresponding to this plateau. The dependence of the analytical signal of 20Si on ONX 0914 concentration was investigated as its concentration ranged between 0.5 and 25 *μ*g mL^−1^, at a constant 20Si concentration of 2.8 *μ*g mL^−1^ and pH 7.4. Time of contact of the 20Si solution with the biosensor was 10 min. The dependence is shown in Fig. 2a. The gradual formation of a plateau is evidence of saturation of the biosensor surface with ONX 0914. A further increase in ONX 0914 concentration does not cause an increase in surface ONX 0914 concentration. On the basis of curve A in Fig. 2, a concentration 15 *μ*g mL^−1^ was selected as the optimal ONX 0914 concentration for further investigations.

### Calibration Curve for 20Si

**Figure 3:**
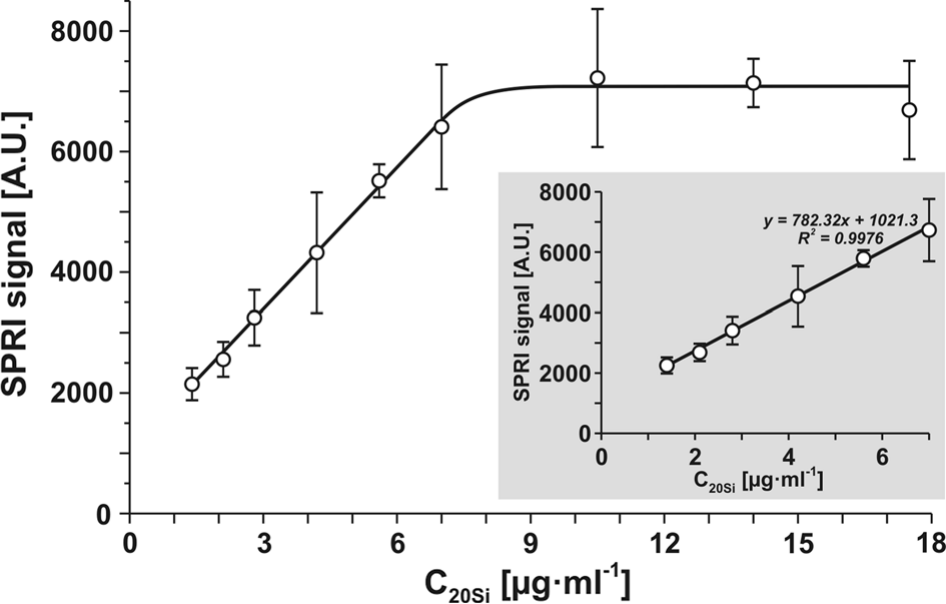
Calibration curve for 20Si with biosensor consisting of ODM linker and ONX 0914 inhibitor as the receptor. Concentration of ONX 0914: 15 *μ*g mL^−1^, pH 7.4. Interaction time: 10 min.

The calibration curve for 20Si determination with a biosensor consisting of ODM linker and the inhibitor ONX 0914 as the receptor was performed in optimized conditions. SPRI measurements were performed in a range between 1 and 15 *μ*g mL^−1^. The results are shown in Fig. 3a. The curve is of typical of Langmuirian type. The plateau of the curve corresponds to saturation of active sites of the biosensor. The initial section of the curve (Fig. 3) is linear and is useful for analytical purposes.

### Specificity of the Developed Biosensor

The specificity of the developed biosensor, especially against 20Sc, is crucial factor for the device’s application. Specificity was investigated at different ratios of 20Si and 20Sc and various 20Si concentrations. Additionally, tolerance of human albumin was checked. The results are shown in Table 3.

**Table 3:** Influence of proteasome 20Sc or human albumin on the determination of 20Si concentration by SPRI biosensor with ONX 0914 as a receptor.

Potential interferent	Ratio 20Si vs. interferent	Added 20Si (*μ*g mL^−1^)	Found 20Si (*μ*g mL^−1^)	Recovery (%)
20Sc	1:1	2.8	2.82 ± 0.44	101
20Sc	1:5	2.8	3.08 ± 0.22	110
20Sc	5:1	7.0	7.31 ± 0.67	104
Albumin	1:1000	2.8	2.94 ± 0.12	105

Confidence limits were calculated for 12 independent measurements for each concentration at a 95% confidence level

20Si, immunoproteasome; 20Sc, constitutive proteasome

It is clear that a 5-time excess of 20Sc has a small, analytically acceptable effect on the 20Si determination. ONX 0914 is an irreversible inhibitor of immunoproteasome. It is an α′β′-epoxyketone which blocks the β5i-subunit 20–40 times more selectively than other active sites of the immunoproteasome.^19^ 20Sc at a lower ratio against 20Si has a negligible effect on the 20Si determination, as does a 1000-fold excess of human albumin. The recoveries and precision of determination, the latter represented by confidence ranges, are well acceptable for analytical purposes. Examples of 20Si determination in real samples are given in a further part of the paper.

### Biosensors Based on Newly Synthesised Receptors

Because the ONX 0914 inhibitor was synthesized for therapeutic purposes, an attempt to synthesized a receptor of simpler chemical structure was undertaken. Apart from the active part necessary for specific 20Sc entrapment, the new receptor was expected to have a carboxylic group for covalent bonding to a linker. The synthesis of 11 receptor candidates, as well as their basic properties and enzymatic activity, were described above under Procedures. Examination of the selectivity of these tripeptides is described below.

### Selectivity of Synthesised Receptors

A crucial factor for the application of newly synthesised potential receptors is their selectivity. Selectivity for 20Si or 20Sc was checked using SPRI measurements. For this purpose, SPRI measurements were performed for 11 newly synthesised potential receptors and for ONX 0914, known to be a selective 20Si inhibitor.^14^ Measurements were performed as described above at three different concentrations of potential receptors (1, 10, 100 *μ*g mL^−1^) at a fixed concentration of 20Si (2.8 *μ*g mL^−1^) or 20Sc (2.8 *μ*g mL^−1^). An increase in the SPRI signal of less than 20% was considered to imply lack of ability to bind 20Si or 20Sc. The obtained results are shown in Table 4.

**Table 4:** The ability of newly synthesised potential receptors and ONX 0914 to bind 20Si or 20Sc.

Potential receptors	Determined substance	
	20Si	20Sc
1	+	+
2	+	+
3	+	+
4	–	–
5	+	–
6	–	+
7	+	+
8	+	–
9	–	+
10	+	–
11	+	–
ONX 0914	+	–

Commercially available proteasome was considered as 20Sc

+, bound; −, lack of bonding

Tripeptides 1,2,3 and 7 are able to bind both 20Si and 20Sc, like the PSI inhibitor, while tripeptide 4 does not capture any form of 20S. Tripeptides 6 and 9 exhibit selectivity to 20Sc. However, the results of preliminary attempts to use them as 20Sc-specific receptors were discouraging. The expected selective inhibition of 20Si is exhibited by tripeptides 5, 8, 10 and 11, as well as by ONX 0914, which is known as selective 20Si inhibitor.

### Optimization of Receptor Concentration

The dependence of the analytical signal of 20Si on receptor concentration was investigated with a biosensor having receptor 5 within a receptor concentration range between 0.5 and 25 *μ*g mL^−1^, at a constant 20Si concentration of 2.8 *μ*g mL^−1^ and pH 7.4. The dependence is shown in Fig. 2b. Due to the same mass of the other inhibitors, it was presumed that the curves for inhibitors 8,10 and 11 have similar shape and plateau. The gradual formation of a plateau is evidence of saturation of the biosensor surface with the receptor. A further increase in the concentration of receptor 5 does not cause an increase in the receptor surface concentration. It should be noted that the analytical signal obtained with the use of receptor 5 is lower than when the inhibitor ONX 0914 is used as the receptor. 15 *μ*g mL^−1^ was selected as the optimal concentration for further investigation on the basis of curves in Fig. 2.

### Calibration Curves of 20Si with Biosensors Having Receptors 5, 8, 10 and 11

**Figure 4:**
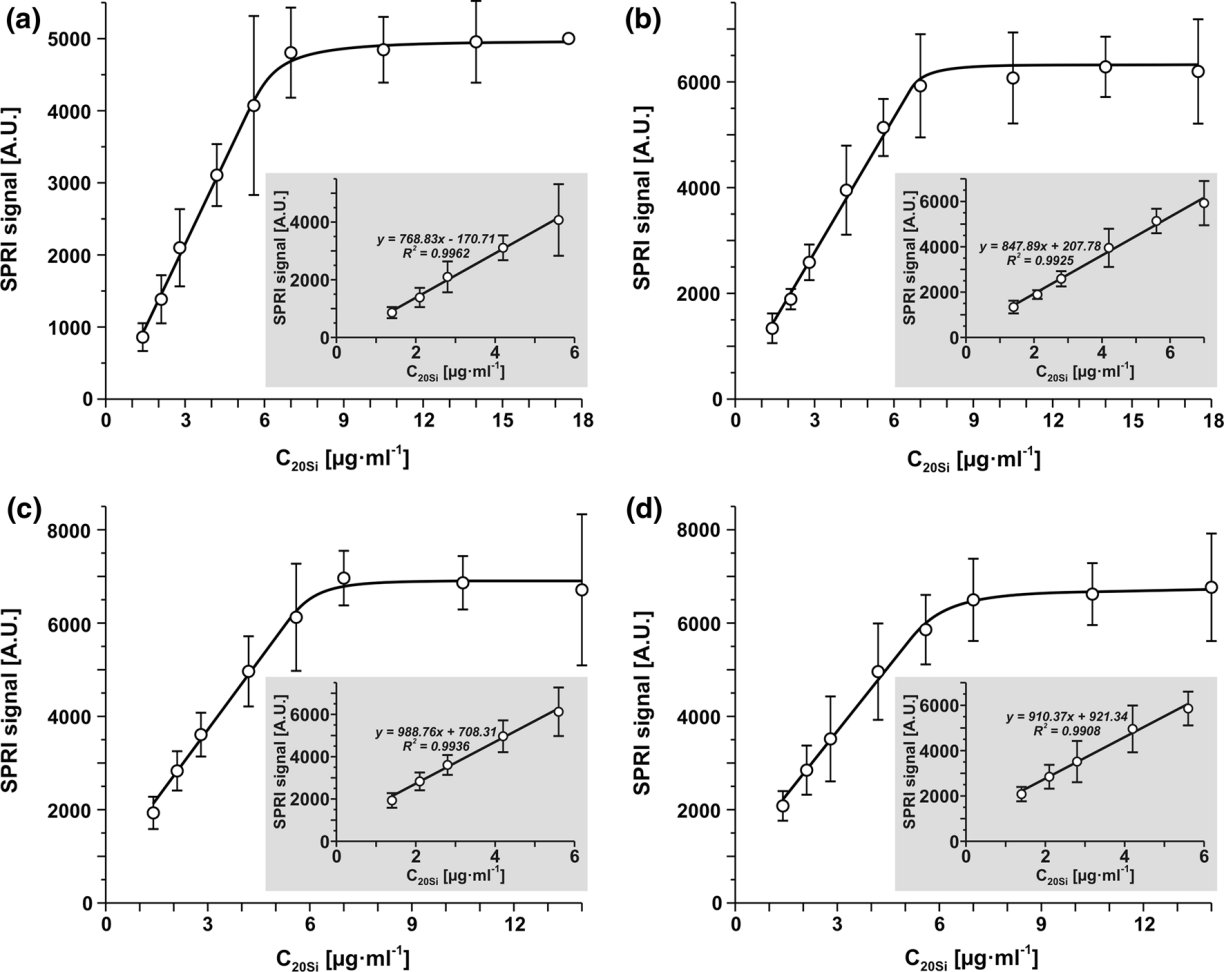
Calibration curves for biosensors with receptors: 5 (a), 8 (b), 10 (c) and 11 (d), pH 7.4, interaction time: 10 min.

The calibration curves for 20Si determination with four biosensors consisting of cysteamine linker and receptors 5, 8, 10 and 11 were obtained in optimized conditions. SPRI measurements were performed within a range between 1 and 15 *μ*g mL^−1^. Results are shown in Figs. 4a–4d for receptors 5, 8, 10 and 11 respectively. Generally, all four calibration curves have the same shape, with straight initial sections which are useful for analytical purposes. All straight sections have an R^2^ value close to 1. The linear sections of the curves for receptors 5 and 8 begin from 0, while the remaining two have intercepts similar to the calibration curve for the ONX 0914 inhibitor.

### Specificity of the Developed Biosensors with Receptors 5, 8, 10 and 11

This investigation is crucial for the determination of 20Si in the presence of 20Sc. Series of model solutions containing both 20Si (2.8 *μ*g mL^−1^) and 20Sc (2.8 *μ*g mL^−1^) were determined using four developed biosensors specific for 20Si. The results are shown in Table 5. The confidence limits of the results are a measure of measurement precision.

**Table 5:** Influence of proteasome 20Sc (2.8 *μ*g mL^−1^) on the determination of 20Si concentration by SPRI biosensors with receptors 5, 8, 10 and 11.

Receptor	Ratio 20Si vs. 20Sc	Added 20Si (*μ*g mL^−1^)	Found 20Si (*μ*g mL^−1^)	Recovery (%)
5	1:1	2.80	2.97 ± 0.40	106
8	1:1	2.80	2.88 ± 0.44	103
10	1:1	2.80	2.89 ± 0.60	103
11	1:1	2.80	2.99 ± 0.47	107

Confidence limits were calculated for 12 independent measurements for each concentration at a 95% confidence level

The results show the very small influence of 20Sc on the results of 20Si determination. Thus, the developed biosensors can be used for selective 20Si determination. The precision of the measurements is acceptable. The results are very similar to those obtained with biosensors based on the ONX 0914 inhibitor.

### Determination of 20Sc in the Presence of 20Si

**Figure 5:**
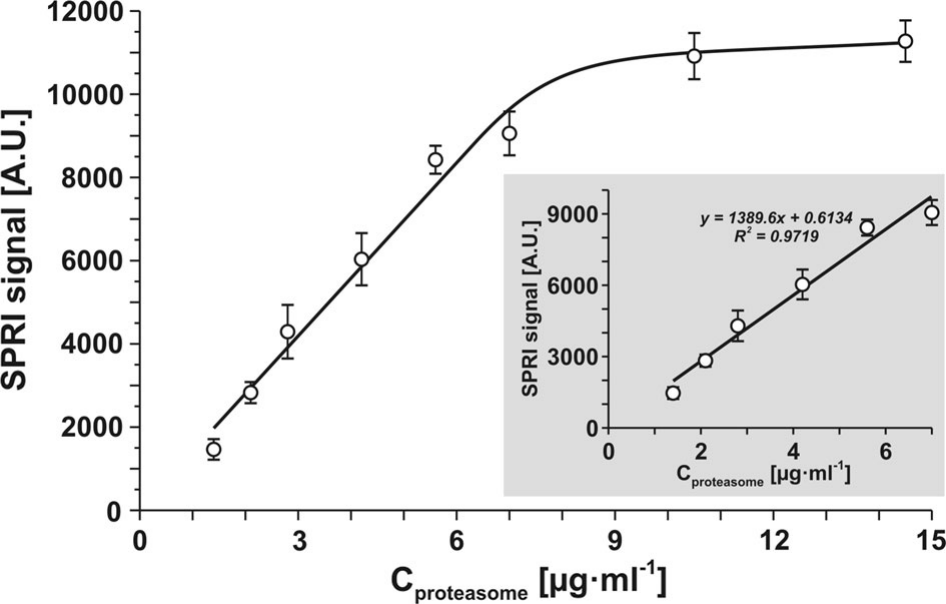
Calibration curve of total 20Sc and 20Si with biosensor based on PSI receptor (25 *μ*g mL^−1^), pH 7.4.

Apart from direct application of the newly synthesized tripeptides and ONX 0914 inhibitor for 20Si determination, these compounds can be used indirectly for the determination of 20Sc. A biosensor containing inhibitor PSI can be used for this purpose. This biosensor is selective for the total 20S concentration i.e. the sum 20Si and 20Sc.^17^ Selective blocking of 20Si should result in specific 20Sc determination. For this purpose, a biosensor for selective 20S determination based on PSI inhibitor was made, and a calibration graph was constructed as shown in Fig. 5.

A series of measurements was performed to examine the possibility of 20Sc determination in the presence of 20Si blocked by inhibitor ONX 0914 or tripeptides 5, 8, 10 and 11 used as 20Si inhibitors. The experiments were performed at a 1:1 ratio of 20Sc and 20Si. The results in Table 6 show that the concept can be realized successfully and that 20Sc can be selectively determined. Generally, the recoveries of 20Sc in this experiment can be acceptable. It should be noted that independent determination of 20Si and 20Sc and comparison of the results with independent determination of total 20S is the most credible option.

**Table 6:** Influence of proteasome 20Si (2.8 *μ*g mL^−1^) on the determination of 20Sc concentration by SPRI biosensors using 5, 8, 10, 11 and ONX 0914 inhibitors as blocking solutions.

Receptor	Ratio 20Sc vs. 20Si	Added 20Sc (*μ*g mL^−1^)	Found 20Sc (*μ*g mL^−1^)	Recovery (%)
5	1:1	2.80	2.93 ± 0.36	105
8	1:1	2.80	2.60 ± 0.35	93
10	1:1	2.80	2.69 ± 0.33	96
11	1:1	2.80	2.60 ± 0.53	93
ONX 0914	1:1	2.80	2.55 ± 0.54	91
PSI	1:1	2.80	5.31 ± 0.45*	95

Confidence limits were calculated for 12 independent measurements for each concentration at a 95% confidence level

*Expected total 20Sc and 20Si concentration is equal to 5.60

### Determination of 20Si and 20Sc Concentration in Biological Samples

In order to verify the developed methods for 20Si and 20Sc determination, two series of measurements of the concentration of these analytes in blood plasma were performed. The first series consisted of four samples of human blood plasma of healthy donors. 20Si was determined using the five developed biosensors separately. 20Sc was determined with the use of a biosensor based on PSI inhibitor. 20Si in the samples was inactivated by the addition of inhibitors at a concentration of 15 *μ*g mL^−1^. ONX 0914, 5, 8, 10 and 11 were used separately in each measurement series. The total concentration of 20 Si and 20Sc was also determined i.e. without inhibitor. The results are shown in Table 7.

**Table 7:** A comparison of average concentrations: (a) immunoproteasome determined on the synthesized inhibitors and ONX 0914, (b) proteasome determined on the receptor PSI with blocking immunoproteasome, (c) proteasome determined on the receptor PSI without blocking immunoproteasome.

Inhibitor no as receptor	5	8	10	11	ONX 0914
(a) Immunoproteasome (20Si), C = (*μ*g mL^−1^)					
Healthy donors, n = 4	1.19 ± 0.32	1.35 ± 0.47	1.19 ± 0.32	1.32 ± 0.49	1.42 ± 0.45
Patients with acute leukemia, n = 5	41.05 ± 10.18	46.78 ± 12.17	44.11 ± 10.28	44.23 ± 10.45	39.36 ± 5.46
(b) Proteasome (20Sc) (with blocking immunoproteasome), C = (*μ*g mL^−1^)					
Inhibitor no as blocker	5	8	10	11	ONX 0914
Healthy donors, n = 4	1.64 ± 0.84	1.17 ± 0.61	2.08 ± 0.51	1.76 ± 0.59	1.67 ± 0.43
Patients with acute leukemia, n = 5	10.55 ± 4.73	11.43 ± 8.32	8.21 ± 0.42	9.54 ± 0.53	8.90 ± 4.89
(c) Proteasome (20S) (without blocking immunoproteasome), C = (*μ*g mL^−1^)					
Healthy donors, n = 4	2.80 ± 0.70				
Patients with acute leukemia, n = 5	43.00 ± 10.40				

Confidence limits were calculated for 12 independent measurements for each concentration at a 95% confidence level

The second series was performed with five blood plasma samples from acute leukemia patients. The samples were diluted with PBS buffer so as to fit the linear section of the calibration graph. The measurements were performed as described above. The results are shown in the lower part of Table 7. Each of the blood plasma samples was subjected to determination 12 times. The confidence limits of the results reflect the variation of concentration in particular samples as well as the precision error.

No significant difference between the measurements of 20Si concentration with each of the five developed biosensors is observed, or in 20Sc determination using the newly synthesized tripeptides or ONX 0914 as 20Si inhibitors. Each of these tripeptides can be used equivalently in place of ONX 0914 for analytical purposes. The total 20Si and 20Sc concentrations in blood plasma are generally in agreement with independent measurement of the sum of 20Si and 20Sc.

It is clear that in the case of patients with acute leukemia, 20Si is the major form of proteasome and its concentration is highly elevated compared with the level of 20Si in the blood plasma of healthy subjects. In the case of healthy donors, the two proteasome forms occurs at approximately equal concentrations.

### Advantages and Limitations of the Developed Methods

The five developed direct methods for circulating 20Si determination offer an alternative to the ELISA method, which is the only available quantitative method to date. In contrast to ELISA, these methods are direct and label-free. All five methods are equivalent in terms of analytical characteristics, i.e. selectivity, linear response range, precision and recovery. A certain advantage over the other methods is demonstrated by those using receptors 5 and 8, because the linear sections of their calibration graphs have practically no intercepts (see Fig. 4). The similarity of the analytic characteristics of the four methods based on newly synthesized receptors is due to the fact that these receptors are isomers (see Table 1). Surprisingly, the method using ONX 0914 as the receptor exhibits very similar analytical characteristics, despite the difference in chemical structure and in the manner of immobilization on the biosensor surface (hydrophobic interaction vs. covalent bonding for the other receptors). A certain limitation of all of the developed direct methods is the relatively narrow range of linear response, comprising approximately a half of an order of magnitude. This limitation can be overcome by the dilution of samples with buffer.

The five developed indirect methods for constitutive 20S determination exhibit poorer analytical characteristics than the developed methods for 20Si determination. Recoveries are mediocre even under model investigation; the worst is for the method using ONX 0914. However, these methods are unique because they are the first available methods for 20Sc determination. (It should be noted that 20S concentration, i.e. 20Si and 20Sc, is frequently called ‘constitutive 20S’.) The lack of alternatives makes these methods useful.

Experiments with blood plasma confirm the applicability of all five direct and five indirect methods for simultaneous determination of 20Si and 20Sc. Cross-examination of the results (Table 7) by independent determination of 20S concentration (i.e. 20Si + 20Sc) show that the sum of 20Si and 20Sc determined separately exceeds the results of independent measurements by approximately 10% in the case of healthy donors and approximately 20% in the case of patients with leukemia. The reason for this difference may be the unequal response of 20Si and 20Sc in independent measurement (the determination is calibrated with 20Sc). Certainly, these few experiments with blood plasma are insufficient for conclusions to be drawn concerning leukemia; they demonstrate only the applicability of the newly developed analytical tools.

## Conclusions

We have developed five biosensors usable with the SPRI technique, suitable for specific 20Si determination in the presence of 20Sc i.e. the other form of proteasome. One of these biosensors contains commercial ONX 0914 inhibitor bound to the biosensor via an ODM linker by hydrophobic forces. Four other biosensors contain newly synthesized receptors covalently bound to the biosensors via a cysteamine linker. These four receptors were selected from 11 newly synthesized potential receptors candidates. All five developed biosensors have similar characteristics in terms of selectivity, recoveries, precision and dynamic response ranges.

The same substances used as receptors were applied for indirect determination of 20Sc. Here a biosensor with inhibitor PSI was used, such a biosensor cannot distinguish between 20Si and 20Sc. However, 20Si was inactivated by the inhibitors previously used as receptors. Satisfactory analytical characteristics were obtained for all five versions of 20Sc determination. All five new constructed biosensors were used for 20Si determination in blood serum samples, as well as for 20Sc determination after blocking of 20Si. Both 20S proteasome types were determined in the blood plasma of patients with acute leukemia and of healthy donors. 20Si was the major type of 20S proteasome, and its level was more than one order of magnitude higher than in the healthy donors.
